# Automated Wound Image Segmentation: Transfer Learning from Human to Pet via Active Semi-Supervised Learning

**DOI:** 10.3390/ani13060956

**Published:** 2023-03-07

**Authors:** Daniele Buschi, Nico Curti, Veronica Cola, Gianluca Carlini, Claudia Sala, Daniele Dall’Olio, Gastone Castellani, Elisa Pizzi, Sara Del Magno, Armando Foglia, Massimo Giunti, Luciano Pisoni, Enrico Giampieri

**Affiliations:** 1 Department of Physics and Astronomy, University of Bologna, 40127 Bologna, Italy; 2 Department of Veterinary Medical Sciences, University of Bologna, 40064 Ozzano dell’Emilia, Italy; 3 Department of Medical and Surgical Sciences, University of Bologna, 40138 Bologna, Italy

**Keywords:** deep learning, image segmentation, wound healing, transfer learning, active learning, dog, cat, open wound management

## Abstract

**Simple Summary:**

Appropriate wound management shortens healing times and reduces management costs, benefiting the patient in physical terms and potentially reducing the healthcare system economic burden. Artificial intelligence techniques could be used to automate the process of wound healing assessment, easing the effort required by clinicians and removing the inherent subjectivity of the evaluation. However, the training of artificial intelligence models relies on the availability of large datasets of carefully annotated data. The annotation of medical data is a time consuming and expensive process which requires the supervision of high-expertise professionals. In this work, we introduced a novel pipeline for the segmentation of pet wound images, using an advanced training strategy able to minimize human intervention for both the image annotation and wound segmentation. We implemented our solution in a novel mobile app, providing a valuable tool for pet wound treatment and a methodological approach for the generation of large image-segmentation datasets.

**Abstract:**

Wound management is a fundamental task in standard clinical practice. Automated solutions already exist for humans, but there is a lack of applications regarding wound management for pets. Precise and efficient wound assessment is helpful to improve diagnosis and to increase the effectiveness of treatment plans for chronic wounds. In this work, we introduced a novel pipeline for the segmentation of pet wound images. Starting from a model pre-trained on human-based wound images, we applied a combination of transfer learning (TL) and active semi-supervised learning (ASSL) to automatically label a large dataset. Additionally, we provided a guideline for future applications of TL+ASSL training strategy on image datasets. We compared the effectiveness of the proposed training strategy, monitoring the performance of an EfficientNet-b3 U-Net model against the lighter solution provided by a MobileNet-v2 U-Net model. We obtained 80% of correctly segmented images after five rounds of ASSL training. The EfficientNet-b3 U-Net model significantly outperformed the MobileNet-v2 one. We proved that the number of available samples is a key factor for the correct usage of ASSL training. The proposed approach is a viable solution to reduce the time required for the generation of a segmentation dataset.

## 1. Introduction

Wound healing in both human and animal treatments is a complex and multidisciplinary problem [1]. The importance of precise and efficient wound assessment is helpful to improve diagnosis in acute wounds and to increase the effectiveness of treatment plans for chronic wounds [2]. The increasing usage of digital procedures in medicine is encouraging clinicians to explore new solutions to improve clinical practice and patient treatments [3,4]. Smartphone cameras provide valid and ready-to-use solutions for the acquisition of high-quality imaging data [5,6], also offering enough computational power for their real time analysis.

In veterinary medicine, as well as in humans, automated technology for wound management could help to quickly assess the severity of an open wound that may require prompt intervention in acute injuries [7,8,9]; likewise, it may objectify the state of the wound at the time of clinical presentation. Automated solutions for wound management may help in the monitoring of the subsequent wound healing process.

There are only a few AI-based solutions focusing on animal wound management, yet the global animal wound care market size was valued at USD 960.3 mln in 2020 (ref. https://www.grandviewresearch.com/industry-analysis/animal-wound-care-market, accessed on 6 March 2023). Current wound modelling studies mainly focus on laboratory mice, measuring the wound size as a key parameter [10]. Despite the growing literature on performing automated wound management applied to human patients, there is still a general lack of interest in the adaptation of the same techniques on the animal counterpart. Those automated solutions usually leverage the power of artificial neural networks. However, the deployment of these models relies on the availability of a large amount of data and manually annotated samples [11].

Features such as the size and shape of the wound areas represent fundamental quantities for the monitoring of the wound healing status and the consequent prognosis of the patient [12]. The introduction of automated solutions to address the feature extraction task could speed up the standard clinical practice and provide a standardized method of wound severity stratification. The extraction of quantitative features able to characterize the lesion in exam starts from a precise identification of the wound region of interest, i.e., the wound segmentation [13,14]. Automated wound segmentation on humans has been addressed via the development of ad hoc Convolutional Neural Network (CNN) [15,16,17] models. Equivalent solutions for pet images are still missing in the literature, probably due to poor data for the training of deep learning models.

The training of an artificial neural network usually needs supervision, but it is generally difficult to obtain great quantities of manually annotated data in clinical settings. In particular, the manual segmentation of images could, indeed, be extremely time consuming for large datasets. However, when available, manual segmentation by human experts could further suffer from imperfections, mainly caused by inter-observer variability, due to a subjective wound boundaries estimation [18].

Several approaches are able to overcome the problem of large data annotations and consequent image segmentation [19,20]. In the work of Zhou et al. [21], the authors proposed the division of each image into a series of disjointed patches, providing to the clinicians only small portions of the whole image in which to focus their manual annotation. Another interesting attempt by Mahapatra et al. [22] introduced the usage of Semi-Supervised Learning (SSL) in combination with Active Learning (AL) on Crohn’s disease; starting from only a few labelled samples, the predictions of the automated model were iteratively refined by experts in different rounds of training. Following the same training strategy, we discussed in our previous work [23] an application to human wound image segmentation using a deep learning model, obtaining state-of-the-art comparable results.

In this work, we extended our previously proposed pipeline to pet images via a transfer learning procedure. We aim to minimize the labeling effort of the clinicians, requiring no starting manual annotation at all. To perform a robust image segmentation without a labelled ground truth, we started from the model that Curti et al. [23] trained on a human wound image dataset.

## 2. Materials and Method

### 2.1. Patient Selection

A set of wound photos of dogs and cats was acquired with 4 different digital cameras at the University Veterinary Hospital of the Department of Veterinary Medical Sciences, University of Bologna, during daily clinical practice. The acquisition procedure was performed from April 2014 to June 2022, with a total of 290 selected images (*PetWound* dataset). The photos included spontaneous wounds of domestic dogs and cats that were brought to the institution for the treatment of the wound, and eventually other concomitant lesions. To be enrolled in the study, images must contain a cutaneous region with an open wound caused by cuts, abrasions, laceration, sores, burns, degloving injuries, avulsion wounds, or dehiscence from previous surgery. The statistics of species involved in the study are reported in Table 1.

Exclusion criteria were set from both a technical and a clinical point of view: some images were rejected because of poor quality, i.e., no sharp definition, inadequate light exposition, etc.; sometimes the kind of injuries represented could not be categorized as proper open wounds (open wound during surgery sutured wounds, and wounds with other surgical implants); some images with bloodstains or red-colored objects in the background were also excluded.

### 2.2. Data Acquisition

The images were acquired during clinical practice by several operators without a standardized procedure. The only guideline was to keep the wound at the center of the image. The aim was to obtain an heterogenous dataset, with different light conditions (illumination and exposition) and variable backgrounds.

All the images were stored in RGB 8-bit JPEG format, with different dimensions according to the device used. The details of the camera devices used for the acquisition are shown in Table 2.

### 2.3. Training Strategy

In this work, we developed an ad hoc Active Semi-Supervised Learning (ASSL) strategy, reproducing the procedure already discussed in our previous work [23]. The ASSL procedure was reiterated for several rounds of training, requiring the clinicians to simply accept or discard the output of the model after each round. We used the model trained on the human wound *Deepskin* dataset without any refinement to produce the initial labels for the pet wound images. This procedure substitutes the manual annotation for the initial round of the ASSL pipeline.

We used a deep CNN U-Net [24] with an EfficientNet-b3 [25] backbone as a model for automated segmentation. At each round of ASSL, the model weights were restored to the original configuration obtained by training on the *Deepskin* dataset, i.e., a human-based wound dataset. The use of a pre-trained configuration of the model weights during ASSL rounds facilitates the learning process, providing a solid starting point for a Transfer Learning (TL) procedure. The TL strategy allows a faster convergence of the model training, as already proved by several authors in image analyses [26,27,28].

The initial set of predicted segmentations was validated by two expert clinicians. For each validation image, the clinicians determined whether the generated segmentation was accurate according to the following binary criteria: (i) the mask must cover the entire wound area; (ii) the mask must cover only the wound area, i.e., the mask must not have holes or spurious parts; (iii) the mask shape must follow the correct wound boundaries. The validated images (and corresponding segmentation masks) which satisfied all the criteria were inserted into the training set as the starting point of the current ASSL training strategy. Ad hoc software was developed to minimize the time required for the validation procedure [29]. The validation was performed, reviewing the segmentations overlayed on the original image displayed on a high-resolution screen and without limits of time for the evaluation.

An analogous pipeline of ASSL in combination with TL was used for the training of a MobileNet-v2 U-Net [30] architecture. The MobileNet-v2 U-Net is a standard candidate for mobile implementations, and it guarantees a faster evaluation of the images with a small number of parameters to tune. The MobileNet-v2 U-Net model was pre-trained on the *Deepskin* dataset (obtaining segmentation performances compatible with the results achieved in our previous work [23]) and used in the ASSL training strategy as described for the EfficientNet-b3 one. The use of a smaller model as a benchmark allows a quantitative measurement of the required model complexity for the wound segmentation task in relation to the number of the available samples: small datasets are commonly analyzed with simpler deep learning models, but there are no clear guidelines about this criterion in combination with ASSL training strategy.

The implementation of the ASSL training strategy aims to progressively increase the number of correctly annotated samples. Starting from a small fraction of the original set of data labelled with the application of TL, we aimed to reach at least 80% of correct segmentations. At each round of ASSL, we randomly split the labelled data into two disjointed sets of images, using 90% of the images for the training and 10% for the performance evaluation.

### 2.4. Training Metrics

The efficiency of the ASSL training was evaluated according to the number of correctly segmented images after clinical evaluation. Additionally, in order to prevent possible overfitting of the model during the training stage, we monitored the efficiency of the model using standard metrics. The number of correct segmentations was evaluated on the totality of the *PetWound* dataset, while the efficiency metrics were quantified on 10% of images used for the performance evaluation.

**Figure 1:**
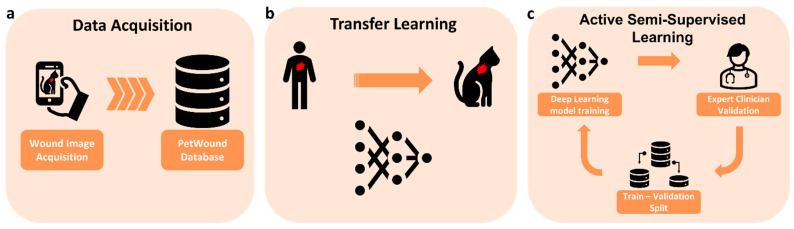
Representation of the pipeline implemented for the training of the EfficientNet-b3 model. (**a**) The images acquired during clinical practice are used as data for the ASSL procedure. (**b**) At the first round, the initial set of labelled images was obtained by the application of the model pre-trained on the *Deepskin* dataset, without any refinement of the model parameters. (**c**) The obtained set of labelled images was used as the kick-start for the ASSL training strategy, performing a TL from the human-based wounds to the pet ones. At each round of training, the model was reset to the initial conditions, i.e., the parameters obtained by the training on the *Deepskin* dataset.

In particular, the F_1_ and IoU metrics used for the performance evaluation are defined as:

(1) $$precision=\frac{TP}{TP+FP}$$

(2) $$recall=\frac{TP}{TP+FN}$$

(3) $$F_{1}=2\times \frac{precision\times recall}{precision+recall}$$

(4) $$IoU=\frac{TP}{TP+FN+FP}$$

where *TP*, *FP*, and *FN* are the True Positive, False Positives, and False Negative scores, respectively. We trained both the models for 100 epochs at each round of ASSL, with Adam optimizer (learning rate of $10^{-5}$). The models were trained minimizing the Binary Focal Loss (*BF*) as loss function:

(5) $$\begin{array}{c} BF_{loss}\left(y_{true},\;y_{pred}\right)=-y_{pred}\alpha {\left(1-y_{pred}\right)}^{\gamma }\log\left(y_{pred}\right)\\ -\left(1-y_{true}\right)\alpha y_{pred}^{\gamma }\log\left(1-y_{pred}\right) \end{array}$$

where $y_{true}\;$and $y_{pred}\;$are the ground truth binary mask and the predicted one, respectively. In our simulations, we used a value of α = 0.25, β = 1, and γ = 2. The data augmentation was restricted only to horizontal and vertical flips. All the simulations were performed using a 64-bit workstation machine (8 GB RAM memory and 1 CPU Intel^®^ i5-8250U CPU, with 4 cores, and a UHD Graphics 620 Intel^®^). A schematic representation of the proposed pipeline is shown in Figure 1.

## 3. Results

### 3.1. Transfer Learning on EfficientNet-b3

The application of the pre-trained EfficientNet-b3 U-Net model on the *PetWound* dataset produced a total of 143 (49% of the whole dataset) correctly annotated images. This core set of images (identified as Round 0 of ASSL procedure) was used as a kick-start for the ASSL training strategy.

The ASSL procedure reached 80% of correctly annotated wound images after five rounds of training. The results obtained by the model along each ASSL round are reported in Table 3, expressed in terms of segmentation metric scores and number of images used for the training/testing of the model. For each round, we also reported the number of correctly annotated images according to the defined criterion.

**Figure 2:**
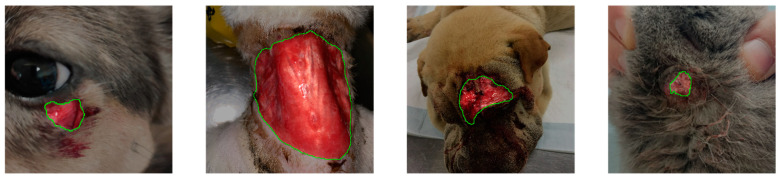
Example of segmentations obtained by the EfficientNet-b3 model at the end of the 5th round of ASSL training. In green, we marked the contours of the segmentation masks produced by the model, highlighting with the alpha level the region of interest identified. The aspect ratio of the images was manually adjusted to be coherent among them.

At the final round of ASSL training, the model achieved an F_1_ score of 0.98 and a corresponding IoU score of 0.96. Examples of the results obtained with *PetWound* images are shown in Figure 2.

### 3.2. Transfer Learning on MobileNet-v2

The application of the pre-trained MobileNet-v2 U-Net model on the raw *PetWound* dataset led to the correct annotation of 113 images (39% of the whole dataset). This core set of images was used to kick-start the ASSL training strategy for a total of four rounds of training. The results obtained by the application of the MobileNet-v2 U-Net model in the ASSL pipeline are reported in Table 4.

We stopped the ASSL training strategy after four rounds of training because the model performances reached a plateau. The MobileNet-v2 U-Net model did not achieve results compatible with the EfficientNet-b3 one, showing a significant gap in terms of corrected segmentation images. Despite the introduction of the TL, the lighter model was not able to generalize on the heterogeneous *PetWound* dataset. These results confirmed the need for a deeper or more complex model for the use of the ASSL training strategy.

## 4. Discussion

In this work, we proposed the application of an ASSL training strategy in combination with TL for the automated segmentation of pet wound images. We confirmed the effectiveness of ASSL in labelling large amounts of data with minimal human effort. The introduction of TL in the training pipeline allowed us to start the ASSL without an initial manually annotated dataset, further reducing the workload required by the clinicians. In the proposed procedure, the evaluation of the experts consists only of an accept/discard method, minimizing the time and the effort required for the generation of high-quality segmentation masks.

The final model obtained with the proposed training pipeline achieved an F_1_ score of 0.98 and an IoU score of 0.96, confirming the goodness of the generated segmentations. As showed in Table 3, the model performances are consistent through the rounds of ASSL training with a slight improvement of the two considered metrics with respect to the values of the initial round. We must consider that the model performance could be positively biased, because at each round we were only including images for which we knew the model performed well in the previous round. However, the positive trend of the correctly segmented images along the ASSL rounds shows that the model was improving its prediction and generalization capabilities. The robustness of our training pipeline is enforced by the fact that the EfficientNet-b3 U-Net model trained on the *Deepskin* dataset also generalized well on the *PetWound* dataset, correctly annotating almost 50% of the available images without any fine-tuning of the model.

The results obtained during the ASSL using the MobileNet-v2 U-Net model were not as good as the ones obtained with the EfficientNet-b3 U-Net one. We argue that the MobileNet-v2 backbone could be not powerful enough to perform this complex segmentation task in the initial stages of ASSL, when the number of available samples is extremely limited. The MobileNet-v2 U-Net was then trained on the final set of annotations produced by the EfficientNet-b3 U-Net model, obtaining good results in terms of segmentation metrics (IoU = 0.90, F_1_ = 0.95 with 72% of corrected segmentations on the whole dataset). This result suggests that a simpler model, such as the MobileNet-v2 one, could need a greater number of training samples to learn the meaningful characteristics of the images in complex contests than the one of this study. This result goes against the classical belief that the optimal number of parameters involved in a deep learning model scales with the quantity of available data: the introduction of TL in the pipeline seems to remedy this problem, providing a sufficiently good starting point for the correct generalization of new data.

To our knowledge, there are no available segmentation datasets of pet wound images; thus, the *PetWound* images we collected, and the corresponding segmentations produced in this work, could be used as a valid starting point for the application of deep learning pipelines in similar studies. An ASSL strategy having the *PetWound* dataset as starting point could easily produce hundreds of other pet wound annotated images with minimal effort.

The main limitations of our dataset are the restricted number of available images and the inclusion of only two different pet species, namely dogs and cats. The expansion of the dataset with wound images of other pet species would be beneficial for the generalizability of the study. On the other hand, a strength of our dataset is the high heterogeneity of the included images, presenting different resolutions, light conditions, and backgrounds, making the dataset suitable for real clinical practice scenarios.

## 5. Conclusions

The aim of this work was to develop a model for the automatic segmentation of pet-wound images, starting with no manually labelled samples and using only TL and ASSL training strategies. The combination of the two training strategies proved their effectiveness in generating large amounts of annotated samples with minimal human intervention. This procedure speeds up the validation procedure by clinicians and it is proven to be a viable solution in medical analyses. This work may represent a starting point for the development of automated wound management in veterinary medicine for clinical and research activities.

We found the EfficientNet-b3 U-Net model, comparing its performances with the lighter MobileNet-v2 U-Net one, to be an optimal deep learning model for the ASSL training strategy. We also demonstrated numerically that the complexity of wound segmentation does not require complex deep learning models, showing comparable performances between the EfficientNet-b3 U-Net and the MobileNet-v2 U-Net architectures when trained on a larger set of annotated images. The inclusion of TL components in the ASSL pipeline, indeed, strengthens the generalization capabilities of the trained models.

The results obtained in this paper stand as a reliable solution to perform correct wound image segmentation. The MobileNet-v2 U-Net performances suggest that the future direction of this field could focus on implementations of smartphone-based technologies.

## Figures and Tables

**Table 1:** Description of the images involved in the study. The number of images is split according to the pet species of the patients. The three rows of the table show the number of initial images collected; the number of images after exclusion of inadequate ones; the different wounds considered starting from the included images, since the same wound could have been acquired at different time points in dogs and cats.

	Dog	Cat	Tot.
N° initial images	301	105	406
N° included images	208	82	290
N° included wounds	130	47	177

**Table 2:** Main specifics of the camera devices used for the acquisition of the photos. The *PetWound* database is composed of images acquired with 4 different camera devices. In detail, there are 3 different smartphone digital cameras and 1 Olympus digital camera.

	Redmi Note 9 PRO	Redmi Note 5	ASUS Z017D	Olympus Imaging CORP u770sW
F-stop	f/1.9	f/1.9	f/2	f/3.5
Exposition	1/33 s	1/25 s	1/100 s	1/15 s
Iso sensibility	ISO-330	ISO-200	ISO 71	ISO-71
Focal distance	5 mm	4 mm	4 mm	4 mm
Focal length	25 mm	24 mm	NA	NA

**Table 3:** Results obtained by the EfficientNet-b3 model in the ASSL training strategy on the *PetWound* dataset. For each round, we reported the number of images used for the training and validation of the model, the number of correctly annotated images, and the segmentation metric scores. We considered as Round 0 the one performed with the model trained only on the *Deepskin* dataset.

	Round 1	Round 2	Round 3	Round 4	Round 5
N° training images	127 (88%)	155 (86.5%)	170 (87.5%)	197 (88.2%)	203 (88.5%)
N° validation images	16 (12%)	24 (13.5%)	24 (12.5%)	24 (10.8%)	24 (10.5%)
N° correct segmentation	179 (62%)	194 (67%)	221 (76%)	227 (78%)	232 (80%)
F_1_ score	0.98	0.98	0.98	0.98	0.98
IoU score	0.95	0.96	0.96	0.96	0.96

**Table 4:** Results obtained by the MobileNet-v2 U-Net model in the ASSL training strategy on the *PetWound* dataset. For each round, we reported the number of images used for the training and validation of the model, the number of correctly annotated images, and the segmentation metric scores. We considered as Round 0 the one performed with the model trained only on the *Deepskin* dataset.

	Round 1	Round 2	Round 3	Round 4
N° training images	97 (86%)	119 (88%)	122 (88.5%)	127 (88.5%)
N° validation images	16 (14%)	16 (12%)	16 (11.5%)	16 (11.5%)
N° correct segmentation	135 (47%)	138 (48%)	143 (49%)	145 (50%)
F_1_ score	0.97	0.92	0.93	0.94
IoU score	0.94	0.85	0.87	0.89

## Data Availability

The data used during the current study are available from the corresponding author on reasonable request. The pre-trained model and parameters used for the image segmentation are available in the Github-repository (https://github.com/Torbidos7/PetWound, accessed on 6 March 2023).
